# Linking the Primary Cilium to Cell Migration in Tissue Repair and Brain Development

**DOI:** 10.1093/biosci/biu179

**Published:** 2014-11-25

**Authors:** Iben Rønn Veland, Louise Lindbæk, Søren Tvorup Christensen

**Affiliations:** Iben Rønn Veland (irveland@bio.ku.dk) is a postdoctoral researcher from the Christensen Lab, at the University of Copenhagen, Denmark, and she studies the role of primary cilia in cell polarization and migration. Louise Lindbæk (llindbaek@bio.ku.dk) is a PhD student in the Christensen Lab, and she studies the function of primary cilia in neurogenesis and brain development. Søren Tvorup Christensen (stchristensen@bio.ku.dfk) is a professor at the University of Copenhagen. He studies how primary cilia coordinate signaling pathways during development and in tissue homeostasis.

**Keywords:** primary cilia, cellular signaling, cell polarity, cell migration, development, tissue repair

## Abstract

Primary cilia are unique sensory organelles that coordinate cellular signaling networks in vertebrates. Inevitably, defects in the formation or function of primary cilia lead to imbalanced regulation of cellular processes that causes multisystemic disorders and diseases, commonly known as ciliopathies. Mounting evidence has demonstrated that primary cilia coordinate multiple activities that are required for cell migration, which, when they are aberrantly regulated, lead to defects in organogenesis and tissue repair, as well as metastasis of tumors. Here, we present an overview on how primary cilia may contribute to the regulation of the cellular signaling pathways that control cyclic processes in directional cell migration.

**Cell migration is defined by the orchestrated** movements of cells in particular directions to specific locations at specific points in time and is fundamental for immune responses, tissue homeostasis, and many developmental and morphogenic processes, such as gastrulation, neurulation, and organogenesis. Consequently, the impairment or enhancement of cell migration leads to severe developmental disorders and diseases, including intellectual disability, vascular disease, and defective immune responses, as well as cancer mortality due to metastasis, in which malignant cells invade the surrounding tissue (Cordeiro and Jacinto 2013, Takahashi et al. 2013, Valente et al. 2014, Wells et al. 2013). Cell migration is generated by cyclic processes that rely on tight regulation and continuous plasticity of the cytoskeleton, as well as a repositioning of cellular organelles, including Golgi, the centrosome, and the nucleus in the polarized cell (Insall and Machesky 2009, Etienne-Manneville 2013, Holcomb et al. 2013, Maninová et al. 2014). The Golgi apparatus resides in close proximity to the centrosome. During quiescence (growth arrest), the mother centriole constitutes a basal body for the formation of a primary cilium, which protrudes from the cell surface into the extracellular environment (figure 1).

**Figure 1.**
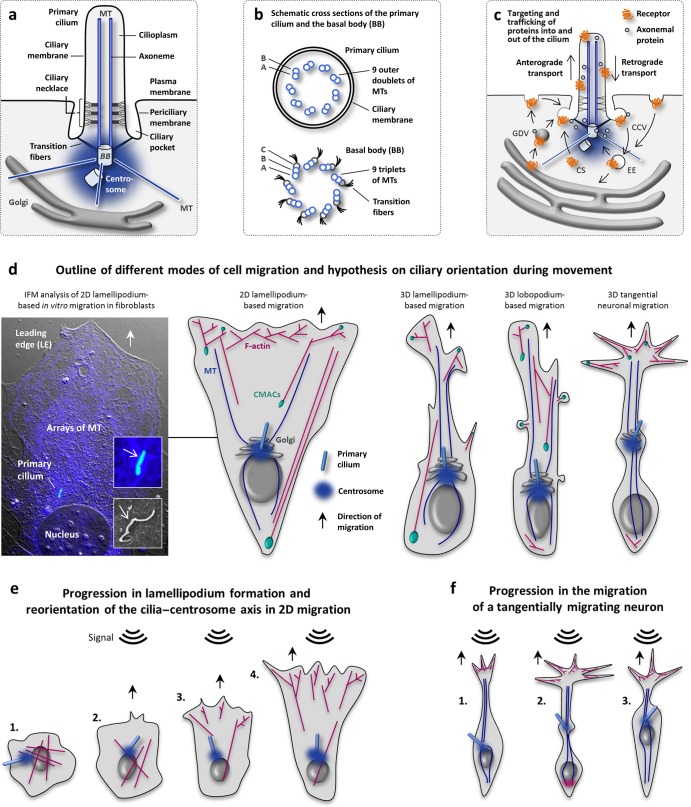
Outline of the primary cilium and modes of cell migration. (a) Primary cilia are microtubule (MT)-based, solitary organelles that emanate from the centrosomal mother centriole (basal body, BB) at the cell surface during growth arrest in most vertebrate cell types. An invagination at the cell membrane forms the ciliary pocket, in which transition fibers aid the docking of the BB to the periciliary membrane. The ciliary necklace is part of the ciliary pore complex, which functions as a diffusion barrier for the trafficking of proteins into and out of the cilium. The Golgi apparatus lies in close proximity to the base of the primary cilium. (b) The axoneme of the cilium comprises nine outer doublets of MTs that extend from the A and B subfibers of triplet MTs in the basal body. (c) The periciliary membrane is a site for the exocytosis and clathrin-dependent endocytosis in the dynamic trafficking of membrane and axonemal proteins into and out of the cilium across the ciliary pore complex. (d) Hypothesis on cellular morphologies and ciliary orientation in various migration modes. The orientation of the primary cilium toward the leading edge in 2D lamellipodium-based migration is illustrated with an immunofluorescence microscopy image of a fibroblast migrating into the wound of a scratch assay. The primary cilium (the open arrow) and arrays of microtubules projecting from the centrosome are stained with anti-acetylated α-tubulin (in blue). Source: Adapted with permission from Christensen and colleagues (2013). The lower insert shows the fibroblast primary cilium protruding from the ciliary pocket by scanning electron microscopy analysis. Micrograph: Johan Kolstrup, Peter Satir. (e) Fibroblast (1) lamellipodium formation and reorientation of the cilia–centrosome axis. Initial posterior positioning of the nucleus (2) is followed by reorganization of the actin cytoskeleton, and reorientation of the cilia–centrosome axis (2). Migration is initiated as the cilium points forward (3), and actin forces the membrane in the direction of the signal (4). (f) Tangentially migrating neurons extend a leading process in the direction of migration (1). Movement is a two-step process, consisting of pushing (2) and pulling (3) forces. First, a swelling occurs containing the Golgi–centrosome axis, including the primary cilium. This event is accompanied by pushing forces of actin or myosin at the posterior end (2). Simultaneously, pulling forces mediated by anterior MTs result in nuclear translocation (3). During nuclear translocation, the primary cilium may transiently be resorbed (Baudoin et al. 2012). Abbreviations: CCV, clathrin-coated vesicle; CMACs: cell-matrix adhesion complexes; CS, centriolar satellites; EE: early endosome; GDV, ­Golgi-derived vesicle. Color image available online at http://bioscience.oxfordjournals.org.

To functionally adapt to the migratory state in many morphogenic, homeostatic, and metastatic processes, apicobasally polarized tissue cells undergo epithelial or endothelial-to-mesenchymal-transition (EMT), whereby polarity is lost via turnover of adherens and tight junctions. In mesenchymal cell types, such as fibroblasts, the cyclic processes in cell migration include membrane protrusion and substrate adhesion in the direction of movement—that is, at the leading edge of the cell—in concert with cell contraction, detachment, and retraction of the rear end. These steps are continuously repeated during the course of the migratory event and are preceded by the establishment of the cell's anterior–posterior polarity, which is a prerequisite for directionality in cell migration (Etienne-Manneville 2012, 2013, Bisi et al. 2013, Cordeiro and Jacinto 2013). The term *planar cell polarity* (PCP) usually refers to the polarization of cells within the plane of a tissue, preceding collective cell migration or convergence extension movements, but is also frequently used to describe the anterior–posterior polarity in individually migrating cells. The molecular mechanisms underlying polarity establishment in addition to the nature of cell protrusions can differ vastly between cell types and among their extracellular environments (Doyle et al. 2013).

Polarization occurs in response to positional cues, such as chemical attractants or the disruption of contact with neighboring cells that guide and adjust the cytoskeletal reorganization and the associated transport of proteins and mRNA to the leading edge throughout the migratory process (Etienne-Manneville 2012, 2013, Cordeiro and Jacinto 2013). *In vivo*, cell migration occurs in two dimensions, such as on a basal lamina, or in three dimensions—for example, through connective tissues or between cells during tissue invasion. Different cell types display various morphological characteristics as they migrate (figure 1d). In contrast to fibroblasts, which primarily extend their leading edge as a lamellipodium or lobopodium (figure 1e; Doyle et al. 2013), the stereotypic migrating neuron can form extensive branched and elongated leading edge protrusions (Holcomb et al. 2013). Furthermore, neuronal movement first requires the elongation and stabilization of the leading protrusion and forward motion of the centrosome, Golgi, and endoplasmatic reticulum, followed by nucleokinesis—that is, translocation of the nucleus (figure 1f; Guo and Anton 2014). The directionality, speed, and persistence of cell migration, in addition to EMT, are crucially regulated by a context-dependent series of cellular signaling systems, including transforming growth factor beta/bone morphogenic protein (TGFβ/BMP), receptor tyrosine kinase (RTK), wingless/int (Wnt), hedgehog (Hh), and G-protein coupled receptor (GPCR) pathways. The spatiotemporal polarization and activation of these signaling systems may have diverse but intersecting effects on the regulation of the cytoskeleton and the dynamics of focal adhesions during tissue homeostasis, as well as in developmental processes.

Primary cilia are microtubule (MT)-based organelles that form as a single copy on the surface of most quiescent cells in the human body (figure 1a–1c). These cilia function as cellular sites for mechano-, chemo-, and osmosensation, which regulate cellular processes during embryonic development and in tissue homeostasis (Satir et al. 2010). Accordingly, defects in ciliary assembly, maintenance, or function are associated with a variety of genetic diseases and disorders, such as early embryonic death, congenital heart disease, craniofacial and skeletal patterning defects, polycystic kidney disease, cognitive disorders, anosmia, obesity, and tumorigenesis (Hildebrandt et al. 2011, Koefoed et al. 2014, Valente et al. 2014). Notably, many of the signaling systems that control cell migration are also coordinated by primary cilia, such as Hh, Wnt, TGFβ, and RTK signaling (Goetz and Anderson 2010, Christensen et al. 2012, Clement et al. 2013a, Oh and Katsanis 2013). In this regard, mounting evidence shows that defects in the formation or sensory function of primary cilia are associated with a series of migration-related disorders and diseases. Here, we present an overview of the function of primary cilia in cell migration, with examples of how cilia coordinate cellular signaling pathways and interact with the extracellular matrix (ECM) during migratory responses.

## Regulating the plasticity of the cytoskeleton during migration

In the polarized cell, projections rich in filamentous actin (F-actin), such as filopodia and lamellipodia, promote plasma membrane protrusion at the leading edge. The formation of these structures requires the nucleation, polymerization, and branching of F-actin, in addition to polarized trafficking of protrusion-promoting components to their sites of action (Bisi et al. 2013). For instance, the Rho GTPases RhoA, Rac1, and Cdc42, in addition to a number of their associated guanine nucleotide exchange factors and activator proteins are essential regulators of many aspects of cell motility and are tightly coupled to MT and actin dynamics (Etienne-Manneville 2013). The F-actin-based membrane protrusions are attached to the substrate and anchor the cell to the substratum or ECM through integrin-mediated nascent adhesions and focal complexes, some of which mature into more-persistent focal adhesions. Collectively, these integrin-based transient structures can be referred to as *cell-matrix adhesion complexes* (CMACs), and the tension on the actin cytoskeleton that results from the dynamic formation and turnover of CMACs is the main contributor to the forces driving cellular relocation (Lock et al. 2008, Wolfenson et al. 2011, Jacquemet et al. 2013). Contraction of the cell body is generated by myosin motors on F-actin bundles, and the release of CMACs, in combination with the cross-linking and depolymerization of F-actin, allows for the cell body contraction to be transmitted into retraction of the rear-end (Wolfenson et al. 2011, Cramer 2013).

In addition to this dynamic receptor-mediated anchorage of the cytoskeleton to the ECM, cell migration greatly relies on the modification of the ECM through cellular traction forces and matrix degradation by the actions of membrane-bound and secreted proteases (Vargova et al. 2012, Doyle et al. 2013). Furthermore, CMACs detect ECM composition and rigidity and integrate this into the cell's protrusion rate, migratory speed, and motility mode, while concomitantly transmitting contractile forces from the cell's actin cytoskeleton to the ECM (Lock et al. 2008, Huttenlocher and Horwitz 2011). As an example of the former, fibroblasts can alternate between modes of lamellipodia- and lobopodia-based motility, depending on ECM rigidity and composition (Petrie et al. 2012).

The plasticity of the cytoskeleton and the remodeling of ECM in the vicinity of the cell also rely on local changes in ion concentrations and pH, which are regulated by transport proteins in the cell membrane (Stock et al. 2013). Among these is the sodium–hydrogen ion exchanger isoform NHE1, which is essential for leading edge protrusion and is implicated in several aspects of cell motility, including the regulation of pH and cell volume, in addition to the branching, stabilization, and anchorage of the actin cytoskeleton to the cell membrane (Boedtkjer et al. 2012). Taken together, the actions repeatedly executed by a migrating cell consist of a finely orchestrated plethora of events, all of which rely on the previous events in order to proceed. Various signaling systems partake in the control of these events, including the sensing of chemotactic gradients and the contact between the cell and the ECM. The primary cilium can contribute to this in a number of ways, as will be reviewed in the following sections.

## The primary cilium points in the direction of migration

Scratch assays and similar two-dimensional (2D) *in vitro* migration approaches have been highly instructive in understanding the role of primary cilia in basic cell migration and invasion parameters such as speed, persistence, and polarity (Christensen et al. 2013, McGowan and McCoy 2013). In scratch assays, cells are grown to confluence, followed by the introduction of a fine abrasion with a pipette tip such that cells are able to migrate into the wound space. Alternatively, a controlled, cell-free area can be created by culturing cells with an insert or a barrier prior to monitoring. In many cases, cell cultures are depleted for serum in order to induce growth arrest, which leads to the formation of primary cilia; yet, many cell types spontaneously form cilia in the presence of the serum, albeit in a less synchronized manner (Wheatley et al. 1994). One of the initial events in 2D directed cell migration is the rearward movement of the nucleus in coordination with the repositioning of the centrosome between the nucleus and the leading edge (figure 1d; Maninová et al. 2014). This process depends on the MT cytoskeleton, in addition to F-actin or intermediate filaments (Etienne-Manneville 2013).

In 1977, Albrecht-Buehler published a seminal work on the orientation of primary cilia in 2D cultures of growth-arrested 3t3 fibroblasts (Albrecht-Buehler 1977). In confluent cultures with no cell migration, the ciliary orientation appeared random, whereas the cilia on cells that migrated in subconfluent cultures oriented predominantly in parallel to the culture substrate and to the direction of migration. Albrecht-Buehler (1977) inferred that primary cilia are either passively oriented during cellular displacement or that the cilium acts as a sensor or mechanotransducer, which actively determines the directionality in cell migration. More recent *in vitro* studies (Christensen et al. 2008, Lu et al. 2008, Wu et al. 2009, Schneider et al. 2010, McGowan and McCoy 2013) have confirmed this unique reorientation of the primary cilium at the wound site of migrating cells (figure 1d, 1e), in which the cilium may directly interact with ECM and control the orientation of the centrosome in front of the nucleus. In some cases, the centrosome may localize behind the nucleus in three-dimensional (3D) migrating cells (Maninová et al. 2014), but it remains to be investigated whether these cells are ciliated during the migratory event.

It is now evident that the position and orientation of primary cilia in the extracellular environment crucially define their function in a variety of different cell types and tissues. Prominent examples include the unique placement of primary cilia on the apical surface of apicobasally polarized epithelial cells, as well as the positioning, projection, orientation, and even bending of cilia in deep tissues (Farnum and Wilsman 2011). In this manner, the primary cilium, in conjunction with the centrosome, may well play a significant role in the integration of spatiotemporal cues that guide the cyclic processes of movement and the placement of cells during development and in tissue homeostasis. As follows, the cilium could function as a point of reference for the navigation of cells in an environment of both opposing and synergistic signals, including soluble morphogens and variations in ECM composition, topography, and rigidity. These signals need to be interpreted and integrated by the cell to yield a behavior, directionality, and speed that, together, constitute the migratory response.

Defects in the formation, organization, or function of primary cilia impair the cyclic processes of cell migration (Lu et al. 2008, Tobin et al. 2008, Wu et al. 2009, Schneider et al. 2010, Higginbotham et al. 2012, Jones et al. 2012, Rozycki et al. 2014) and are associated with migration-related disorders in mice, including aberrant neurodevelopment and brain abnormalities (Tobin et al. 2008, Baudoin et al. 2012, Higginbotham et al. 2012, 2013) and abnormal skin wound closure (Schneider et al. 2010), as well as defects in developmental morphogenesis and the repair of damaged corneal epithelium (Blitzer et al. 2011). In the latter situation, tissue repair is maintained by 2D migration of epithelial cells, in which newly formed primary cilia and their associated basal bodies move and orient toward the wound (Blitzer et al. 2011).

## The interplay between primary cilia and RTK signaling during cell migration

The primary cilium has been linked to coordination of RTK signaling (Christensen et al. 2012), which regulates migratory responses during development, as well as wound healing and tissue renewal in mammals. The RTKs include platelet-derived growth factor receptor alpha (PDGFRα), which localizes to primary cilia in various cell types and is activated by its specific ligand, PDGF-AA, in cultures of fibroblasts (Schneider et al. 2005). In scratch assays with growth-arrested and ciliated wild type mouse embryonic fibroblasts (MEFs), PDGF-AA increases the migration speed and the directional movement of the cells, whereas cells with stunted cilia caused by a hypomorphic mutation in *Ift88* (*Tg737^orpk^* MEFs), a gene required for ciliary assembly through intraflagellar transport (Pedersen et al. 2008), display decreased directionality and no PDGF-AA responsiveness (Schneider et al. 2010). Similarly, fibroblasts isolated from Meckel–Gruber syndrome patients with mutations in the TMEM67/Meckelin fail to form primary cilia and have *in vitro* migration phenotypes similar to that of *Tg737^orpk^* MEFs (Barker et al. 2013).

PDGFRα is encoded by a growth-arrest-specific gene, such that receptor levels are upregulated in cultures of fibroblasts after serum depletion and the formation of the primary cilium (Schneider et al. 2005). Notably, receptor expression in growth-arrested *Tg737^orpk^* MEFs is kept at a basal level comparable to that of cycling cells (Schneider et al. 2005), which could indicate that the lack of PDGF-AA responsiveness in cilia-defective cells is due to lower levels of receptor expression. However, activation of the basal levels of PDGFRα was detected neither in cycling wild type cells nor in quiescent mutant cells after stimulation with PDGF-AA. Using micropipettes to generate a PDGF-AA gradient, ciliated wild type MEFs in 2D cultures responded immediately to a PDGF-AA injection and migrated uniformly toward the pipette, whereas *Tg737^orpk^* MEFs did not respond to PDGF-AA and moved around randomly (Schneider et al. 2010). Similarly, time-lapse microscopy has demonstrated that PDGF-AA increased the migration of postnatal lung fibroblasts with primary cilia orienting toward the leading edge (McGowan and McCoy 2013). Together, these findings indicate that primary cilia are involved in the coordination of PDGF-AA-mediated chemotaxis in fibroblasts.

**Figure 2.**
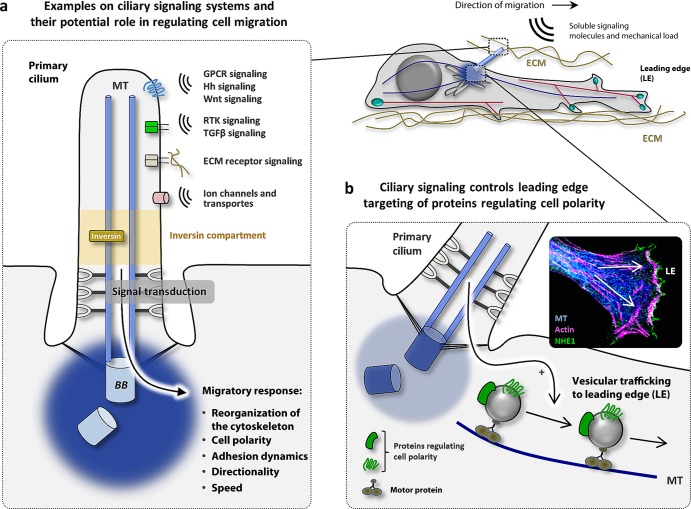
(a) Schematic overview of ciliary signaling systems. The yellow segment of the cilium illustrates the inversin compartment. (b) Specified ciliary signaling pathways may promote (+) the vesicular trafficking and targeting of proteins to the leading edge of the migrating cell to control cell polarity processes. Insert: Localization of the calcium–hydrogen ion exchanger 1 (NHE1, in green) to the leading edge lamellipodium in a migrating fibroblasts. Tracks of microtubules (MT) were stained with anti-α-tubulin (in blue) and F-actin with phalloidin (in magenta). The arrows indicate the direction of NHE1 translocation. Source: Adapted with permission from Clement and colleagues (2013b). Abbreviations: ECM, extracellular matrix; GPCR, G-protein coupled receptor; Hh, Hedgehog; RTK, Receptor tyrosine kinase; TGFβ, transforming growth factor beta; Wnt, wingless/int. Color image available online at http://bioscience.oxfordjournals.org.

The mechanisms by which cilia-mediated signaling is converted into a migratory response are currently not well understood. Questions evidently arise as to how signaling within the cilium, which represents a very small and confined compartment relative to the rest of the cell, and is able to organize the initial steps in cellular adaptation to a gradient of a chemoattractant and how this impinges on the cyclic processes of guided movement. As will be outlined below, there are a number of observations that indicate the functional relationship between primary cilia and the regulation of polarity signaling during the reorganization of the cytoskeleton in cell migration. Furthermore, recent observations have shown that PDGF-AA-mediated migration in scratch assays affects the targeted trafficking and activation of NHE1 to the leading edge of cells facing the wound (Schneider et al. 2009, Clement et al. 2013b), at which the transporter reorganizes the actin cytoskeleton for the formation of a lamellopodium. This implies that ciliary signaling impinges on the coordinated trafficking of specified polarity proteins along microtubules to the leading edge (figure 2) or influences the very positioning of microtubules tracks. Consequently, the PDGF-AA-mediated speed and directionality of cell migration is reduced in NHE1-null fibroblasts or in wild type cells in the presence of inhibitors of NHE1 activity (Schneider et al. 2009). In this scenario, it was suggested that PDGFRα signaling activates the Akt and Erk1/2-Mek1/2-Rsk pathways at the primary cilium. Downstream from here, the Akt pathway may control focal adhesion formation and the initiation of NHE1 translocation to the leading edge, where the transporter is activated. Meanwhile, the Erk1/2-Mek1/2-Rsk pathway may control the spatial organization of NHE1 translocation and incorporation that consequently specifies the direction of the leading edge formation (Clement et al. 2013b). In line with this, the PI3K-Akt and Mek1/2-Erk1/2 pathways have been shown to play crucial roles in the initial events of cell polarization in the 2D migration of growth-arrested normal rat kidney epithelial cells (Bisel et al. 2008, 2013). Although ciliary formation, orientation, and function were not assessed in these studies, the authors showed that Mek1/2-Erk1/2 signaling is required for polarization of Golgi and orientation of the centrosome in front of the nucleus, whereas PI3K-Akt signaling is required for leading edge targeting of the ganglioside GM1 prior to Golgi polarization (Bisel et al. 2008, Bisel et al. 2013).

Additional classes of RTKs have been linked to primary cilia. These include epidermal growth factor receptors and insulin-like growth factor receptor 1 (Christensen et al. 2012, Higginbotham et al. 2013), the former of which localize to the primary cilium of human airway smooth muscle cells (SMCs) and various neuronal subtypes and was suggested to play a part in mechanosensation and the directed cell migration of SMCs (Wu et al. 2009). Evidently, further studies will be needed to clarify how signaling through PDGFRα and other RTKs coordinate ciliary orientation and the cyclic processes of migration and how this is carried out in conjunction with other signaling systems, such as Hh signaling, which specifically affects the persistence but not the speed of cell migration in ciliated postnatal lung fibroblasts (McGowan and McCoy 2013).

## Wnt signaling and polarity processes in cell migration

The Wnt signaling systems, which, in mammals, are initiated by the binding of 1 of 19 Wnt ligands to the family of frizzled receptors (e.g., Fzd3) or context-dependent coreceptors, regulate a series of diverse signal transduction pathways to control embryonic processes during organogenesis and postnatal tissue homeostasis. Canonical Wnt (Wnt/β-cat) signaling operates via β-catenin-mediated gene transcription in cell proliferation and differentiation processes. Noncanonical Wnt responses are β-catenin independent and regulate cell adhesion, motility, and polarization during cell migration, such as in wound repair and during the establishment of PCP. This is predominantly coordinated by disheveled (Dvl) proteins that elicit MT and actin cytoskeletal remodeling via calcium-ion signaling or through a number of Dvl interaction partners and downstream mediators (Gao and Chen 2010, Amin and Vincan 2012, Wynshaw-Boris 2012).

The role of primary cilia in coordinating Wnt signaling is unclear and subjected to much debate due to conflicting results on the requirement of the cilium in organizing individual steps in Wnt signaling during embryonic or fetal development (Ocbina et al. 2009, Wallingford and Mitchell 2011, Oh and Katsanis 2013). However, various Wnt signaling defects are associated with mutations affecting ciliary composition, including the integrity of the basal body and ciliary barriers, and a series of regulatory units in the Wnt pathways localize to the cilia or centrosome axis, including Dvl proteins and β-catenin, as well as components of the β-catenin destruction complex (Oh and Katsanis 2013, Veland et al. 2013, Zilber et al. 2013). Furthermore, Fzd3, which is a receptor for Wnt5a that promotes Wnt/PCP signaling and cell migration, has been localized to the primary cilia of fibroblasts and kidney epithelial cells (Luyten et al. 2010, Veland et al. 2013), although the direct functionality of this in terms of the ciliary control of cell migration remains to be elucidated.

In support of a function of ciliary Wnt/PCP signaling in directional cell migration, inversin/Nephrocystin-2 localizes to a specific subset of the primary cilium, defined as the *inv compartment*, which runs for about 2 micrometers above the ciliary necklace region (figure 2a; Shiba et al. 2009). Inversin acts at the decisive branch point between canonical and noncanonical Wnt signaling by targeting cytoplasmic Dvl for degradation, thus promoting β-catenin turnover and PCP (Simons et al. 2005). MEFs derived from the *invs* mouse (*invs^−/–^* MEFs) display decreased polarization and cell migration in 2D assays, associated with a dispersed accumulation of GSK3β-phosphorylated β-catenin at the ciliary base, elevated canonical Wnt signaling, and aberrant activation and leading edge localization of RhoGTPases (Veland et al. 2013). Although these data indicate ciliary control of Wnt/PCP signaling in the cyclic processes of guided movement, inversin and other ciliary components affecting Wnt signaling also localize to extraciliary domains, such as the leading edge of migrating cells, in which they may contribute to the regulation of cell polarization and migration independently of the cilium (Boehlke et al. 2013, Cui et al. 2013, Werner et al. 2013). Most likely, Wnt signaling coordinates key spatiotemporal events during the cyclic processes of migration through both cilia-dependent and independent pathways.

A number of observations link inversin to PDGFRα signaling in cell migration. Inversin is required for growth-arrest-specific upregulation, as well as leading edge targeting of NHE1, which are both defective in *invs^−/−^* MEFs. This is associated with dysregulated expression, leading edge targeting, and activation of ezrin/radixin/moesin proteins (Veland et al. 2013), which crucially link NHE1 to the actin cytoskeleton and which are required for its activation (Denker and Barber 2002). Moreover, *invs^−/−^* MEFs fail to upregulate *PDGFR*α transcription during growth arrest, which leads to decreased accumulation of PDGFRα in the primary cilium, and, consequently, responsiveness to PDGF-AA and activation of Akt and Mek1/2-Erk1/2 are reduced in these cells (Veland et al. 2007). On the basis of the accumulating data on these signaling pathways, primary cilia, and cell migration, one may envisage the primary cilium as a site for cross-talking between different signaling pathways that regulate the expression, targeting, and activation of key components in polarity processes and directional cell migration. In this regard, it will be interesting to investigate how functional defects in inversin, Wnt, and PDGFRα signaling are interrelated in ciliopathies.

## Primary cilia in neuronal migration and brain development

Neuronal migration during embryonic and fetal development is essential for proper cytoarchitecture and function of the brain (Guo and Anton 2014). Defective migration consequently leads to a series of learning disabilities and cognitive disorders, such as mental retardation and schizophrenia, which have been associated with defects in neuronal primary cilia (Hildebrandt et al. 2011, Valente et al. 2014). Indeed, recent studies have shown a crucial link between neuronal migration and the sensory capacity of primary cilia in the developing brain that may rely on the spatiotemporal dynamics of ciliary receptor composition (Higginbotham et al. 2012, Higginbotham et al. 2013).

Development of the human brain is initiated at gestational week 3, at which the nervous system arises from a uniform sheet of neuroectoderm, forming the neural plate. The neural tube is formed as the ectodermal sheet closes and the brain arises on excessive expansion of the most cranial part of this structure (Tau and Peterson 2010). Gradually, epithelial cells of the neural crest at the dorsal-most side of the neural tube will undergo EMT, deadhere, and migrate as neural progenitors to their site of action. As an example, projection neurons from the dorsal ventricular zone migrate radially, whereas interneurons from regions in the subpallium migrate tangentially into the olfactory bulb or the cortex (Ayala et al. 2007). In radial migration, cells originate in the dorsal part of the ventricular zone and crawl along the extended processes of radial glial cells in order to migrate to various destinations in the cortex. Tangential migration is a mode of nonradial neuronal translocation, predominantly exhibited by interneurons. Primary cilia play a defining role in these events, including the formation of the neural tube and Hh-regulated brain morphogenesis (Tucker and Caspary 2013). The recent focus on ciliopathic syndromes, of which several are associated with cognitive impairment, including nephronophtisis, Joubert syndrome, Meckel–Gruber syndrome, orofacial digital syndrome 1, and Bardet–Biedl syndrome (Valente et al. 2014) have revealed a link between primary cilia and cell migration in the developing brain. As an example, Joubert syndrome, a genetically heterogenous ciliopathy in which all identified genetic mutations are linked to the primary cilium, is characterized by heterotopia in several brain regions and compromised brain tissue architecture (Juric-Sekhar et al. 2012). In addition, although epithelial primary cilia usually project from the apical membrane, some neural crest cells form primary cilia at the basolateral side of the cell layer. This occurs as part of the delamination of these ventricular neuroepithelial cells as they undergo EMT. Because the basolaterally protruding primary cilia are formed adjacent to adherens junctions and project into the ECM (Wilsch-Brauninger et al. 2012), the question arises as to whether the cilium has a unique function in directing cellular orientation in 3D migration through ECM receptors in the primary cilium. The interactions of primary cilia and ECM will be discussed further below, but these findings indicate that the primary cilium and its positioning are tightly linked to the guidance of neuronal cell movements, although the necessity for a primary cilium in controlling neuronal migration varies throughout brain development and between cellular subtypes. In this regard, it is noteworthy that neural crest cells with impaired cilia formation due to the deletion of the integral basal body component, *BBS8*, display marked migratory deficiency, which has been linked to inferior Hh signaling in these cells (Tobin et al. 2008).

## Primary cilia in tangentially migrating neurons

Interneurons migrate tangentially from regions of the subpallium (the ganglionic eminences and the anterior entopeduncular area) through the telencephalon in multiple streams to populate areas of the neocortex, striatum, hippocampus, and olfactory bulb (Ayala et al. 2007, Guo and Anton 2014). In the postnatal and adult brain, cell migration remains. For instance, the positioning of granule neurons in the cerebellum has defining consequences for the development of postural balance and symmetry in movements, which are developed within the first years of life in humans (Ghashghaei et al. 2007), and neuroblasts born in the lateral ganglionic eminence continue to migrate along the rostral migratory stream to the olfactory bulb throughout adulthood (Sawamoto et al. 2006).

The formation of polarized radial glia is the first step in the construction of the cerebral cortex and will serve as a scaffold for the oriented movement of newborn neurons, which climb along glial projections to their target locations, a migratory process that will proceed from gestational week 12 throughout fetal development (Tau and Peterson 2010). Aberrant ciliary signaling resulting from the deletion of the small GTPase Arl13b, which is important for ciliogenesis and the ciliary composition of signaling entities (Caspary et al. 2007), disrupts the formation of a polarized glial scaffold, which leads to neuronal misplacement (Higginbotham et al. 2013). Although primary cilia have not yet been implicated in the migratory process of radially migrating neurons, cilia are indirectly responsible for proper neuronal migration in this scenario and may, in this way, contribute to aberrant neurodevelopment and brain abnormalities.

In a more direct manner, primary cilia were shown to affect the tangential migration of interneurons. Gonadotrophin-releasing hormone (GnRH) neurons in mice migrate a long distance from the nasal pit to the hypothalamic areas of the forebrain during development (Shin et al. 2014), and disruption of ciliary formation by knockout of *Kif3a*, the gene encoding a kinesin subunit required for intraflagellar transport (IFT) and ciliary assembly (Pedersen et al. 2008), leads to an altered topographical distribution of GnRH neurons (Shin et al. 2014). Although there was no apparent change in the total number of migratory neurons across the path, *Kif3a^−/−^* mice displayed an increased number of neurons in the rostral preoptic area of the rostral forebrain (Shin et al. 2014). Kif3a has also been shown to control cortical MT dynamics in MDCK cells (Boehlke et al. 2013), still, these findings indicate a role for the primary cilium in specifying the final location of a subpopulation of GnRH-producing neurons during mouse development.

Indeed, in tangentially migrating neurons, the first migration step is associated with ciliary exposure to the membrane of the leading process. This is followed by a directional change in which the cells switch from tangential migration to radial migration. Guided by ciliary Hh signaling, cells exit their tangential path and continue into the cortical plate (Baudoin et al. 2012). Disruption of ciliary IFT genes and the inhibition of Hh signaling cause medial ganglionic eminence (MGE) cells to retain their tangential paths, which eventually leads to the loss of cortical gamma aminobutyric acid–producing inhibitory interneurons (Baudoin et al. 2012, Higginbotham et al. 2012). These findings indicate that the integrity of primary cilia in the developing cortex is required for Hh-mediated MT-cytoskeletal organization, including the coordination of leading protrusion morphology and the guidance of neurons migrating from the MGE to the cortical plate (Baudoin et al. 2012). As opposed to randomly migrating *Tg737^orpk^* MEFs in 2D cultures (Schneider et al. 2010), disorganized motility of Arl13b-deficient neurons is accompanied by a retarded cell displacement, mainly because of increased pausing between migratory events (Higginbotham et al. 2012). Similarly, both Kif3a- and Ift88/Polaris-deficient MGE cells with defective primary cilia display decreased overall migration speed because of augmented pausing between these events (Baudoin et al. 2012). This correlates with decreased dynamics of ciliary length and movement relative to wild type cells, in which the primary cilia display a range of dynamic morphologies, especially during nonmigrating periods, and appear to be probing the ECM for directional cues (Higginbotham et al. 2012). Using microfluidic chambers for chemotaxis analysis, Arl13b-deficient interneurons were shown to be unable to sense and respond properly to extrinsic guidance cues secreted by dorsal cortical cells, most likely because of alterations in ciliary receptor composition of a series of neuronal GPCRs and RTKs (Higginbotham et al. 2012). Therefore, Arl13b mutant interneurons displayed aberrant activation of receptor-mediated effector molecules in interneuron migration, including increased levels of cyclic adenosine monophosphate and decreased activation of Erk1/2, which supports the conclusion that interneuron primary cilia coordinate the signaling events required for proper corticogenesis.

In summary, these discoveries emphasize the importance of the primary cilium in coordinating neuronal migration during brain development and reflect the necessity of the spatiotemporal dynamics of ciliary receptor composition. The primary cilium presents the cell with the ability to drastically increase local concentrations of receptors, allowing it to respond to subtle environmental changes in the developing brain, and obscuring the system will inevitably have devastating effects on the migration of developing neurons. Further investigations should be directed at elucidating the specific role for primary cilia in neuronal migration during development, as well as in the postnatal brain, which allows us to truly understand the complex circuitry of the mammalian brain and how disorganization may contribute to developmental disorders. This could eventually lead to the therapeutic guidance of newborn cells to damaged sites in the brain.

## Potential roles for interactions between primary cilia and ECM in cell migration

Developmental and regenerative processes highly rely on the cellular deposition of matrix components, as well as the reorganization of existing ECM in connective tissues through local tension forces combined with proteolytic activities. During these events, the dynamic interplay between the cell and ECM via CMACs is crucial in determining the cell's directionality, protrusion rate, and migratory speed. This interplay is largely achieved by integrin signaling in combination with other pathways, including growth factor signaling—for instance, mediated by PDGF and epidermal growth factor receptors—to control cytoskeletal plasticity (Lock et al. 2008, Huttenlocher and Horwitz 2011, Vargova et al. 2012, Jacquemet et al. 2013). Integrin αβ heterodimers, the major CMAC constituents, play a defining role in these processes by forming a link between the actin cytoskeleton and ECM components, such as fibronectin, collagen, and laminin, in addition to various cell surface receptors, depending on the combination of αβ subunits (Huttenlocher and Horwitz 2011).

The primary cilium of numerous matrix-embedded cell types, including fibroblasts, chondrocytes, and SMCs, project from the cell surface into the ECM, while, to various extents, emerging from a ciliary pocket (Farnum and Wilsman 2011). Interestingly, these cilia have been found to harbor different types of ECM receptors (McGlashan et al. 2006, Christensen et al. 2008, Lu et al. 2008, Wu et al. 2009). The proteoglycan NG2 and α2, α3, and β1 integrins localize to chondrocyte primary cilia (McGlashan et al. 2006), and collagens, which serve as ligands for the α3β1 integrins may associate with the ciliary membrane at punctae appearing to correspond to linkage sites between the axoneme and the ciliary membrane (Jensen et al. 2004, McGlashan et al. 2006). ECM receptors have also been documented in the membrane of primary cilia of airway SMCs, which show elaborate accumulation of α2, α5, and β1 integrins (Wu et al. 2009), whereas vascular SMCs display α3 and β1 subunits. In the latter case, ciliary integrin receptor complexes were activated by exposure to collagen (Lu et al. 2008) and inhibition of the β1 integrin–collagen interaction in scratch assays distorted the reorientation of primary cilia toward the wound edge in both airway and vascular SMCs (Lu et al. 2008, Wu et al. 2009). Moreover, the integrin-linked kinase, ILK that serves as a scaffold to link β integrins to F-actin was shown to mediate Hh-induced signaling responses via the primary cilium in neurons, although these are not directly coupled to cell migration (Barakat et al. 2013). Ultimately, tenocytes of the developing chick flexor tendon display a primary cilium, which appears to interact with adjacent cell membranes, perhaps via cell-surface receptors (Poole et al. 1985), although this remains to be identified. The protocadherin Fat4 has previously been localized to primary cilia of the kidney epithelium (Saburi et al. 2008), but although a role for cilia-associated cell-adhesion complexes is plausible in terms of attaching to or sensing other cells along the migratory path, this has, to our knowledge, not been directly addressed.

Elaborate studies have made it clear that the primary cilium is involved in sensing and responding to changes in the ECM, which indicates the potential of counteracting mechanisms induced by the primary cilium; however, the exact means of how the cell uses this information to regulate cell migration remain unknown. Given the intricate role of integrin signaling in controlling directionality and migratory speed (Huttenlocher and Horwitz 2011), it seems likely that ECM receptors in the primary cilium not only sense mechanical changes but also assist in guiding the migrating cell through the ECM, thereby influencing morphogenic processes and wound healing. However, the interactions between the primary cilium and the ECM remain to be investigated during migratory processes, and it will be interesting to follow studies in both 2D and 3D assays.

## Conclusions

The primary cilium may function as a point of reference by organizing the spatiotemporal positioning of a cell in three dimensions during tissue and organ development, as well as in regenerative processes. A migratory response crucially relies on the coordination of many different signaling systems, and specific receptors may become localized to the primary cilium to coordinate the cyclic processes of cell movement, including those responsible for regulating polarity processes and the reorganization of the cytoskeleton, interaction with the ECM, and chemotactic signaling. In this regard, the dynamics of formation, orientation, and length of the primary cilium may serve as a switch by which the cell can control its responsive potential during migratory events. Interneurons have been reported to repeatedly display and remove their primary cilium during their stepwise centrosome or Golgi translocation and nucleokinesis (Baudoin et al. 2012), and corneal epithelial cells uniquely assemble a cilium in response to tissue injury that requires a migratory response (Blitzer et al. 2011). Moreover, TGFβ signaling, which is partly coordinated through clathrin-dependent endocytosis at the base of primary cilia (Clement et al. 2013a) can induce both EMT during heart development (Ezratty et al. 2011) and cilium-dependent differentiation of myofibroblasts from mesenchymal and epithelial precursors. As part of the latter, in scratch assays, primary cilia are resorbed in wound-proximal cells, rendering them insensitive to PDGF-AA and Sonic Hh ligands during their migratory response *in vitro* (Rozycki et al. 2014). The exact mechanisms by which the primary cilium controls persistent directionality in cell migration are currently unknown but have been hypothesized to involve trafficking along stable MTs extending from the centrosome, in which the ciliary basal body is anchored (Clement et al. 2013b). Future research should be directed at understanding how the primary cilium *in vivo* acts as a signaling hub that coordinates the cross-talking between signaling pathways that are activated by chemotactic stimuli on one hand and those triggered by ECM interactions on the other to control migration-dependent processes throughout developmental and in regenerative processes. Furthermore, it will be important to understand how these signaling pathways may either depend on or exploit the primary cilium to control the cyclic processes of cell movement.
